# Effects of temporarily suspending low-dose methotrexate treatment for 2 weeks after SARS-CoV-2 vaccine booster on vaccine response in immunosuppressed adults with inflammatory conditions: protocol for a multicentre randomised controlled trial and nested mechanistic substudy (Vaccine Response On/Off Methotrexate (VROOM) study)

**DOI:** 10.1136/bmjopen-2022-062599

**Published:** 2022-05-03

**Authors:** Abhishek Abhishek, RJ Boyton, Áine McKnight, Laura Coates, James Bluett, Vicki S Barber, Lucy Cureton, Anne Francis, Duncan Appelbe, Lucy Eldridge, Patrick Julier, Nicholas Peckham, Ana M Valdes, Ines Rombach, Daniel M Altmann, Jonathan Nguyen-Van-Tam, Hywel C Williams, Jonathan Alistair Cook

**Affiliations:** 1 Academic Rheumatology, University of Nottingham, Nottingham, UK; 2 Department of Infectious Diseases, Imperial College London, London, UK; 3 Lung Division, Royal Brompton and Harefield Hospitals, London, London; 4 Blizard Institute, Centre for Genomics and Child Health, Queen Mary University of London, London, UK; 5 NDORMS, University of Oxford, Oxford, UK; 6 Manchester Academic Health Science Centre, Manchester, UK; 7 The University of Manchester, Manchester, UK; 8 Oxford Clinical Trials Unit, Department of Orthopaedics Rheumatology and Musculoskeletal Sciences, University of Oxford Nuffield, Oxford, UK; 9 Department of Immunology and Inflammation, Imperial College London, London, UK; 10 Population and Lifespan Health, University of Nottingham, Nottingham, UK

**Keywords:** COVID-19, rheumatologY, clinical trials

## Abstract

**Introduction:**

It is unknown if a temporary break in long-term immune-suppressive treatment after vaccination against COVID-19 improves vaccine response. The objective of this study was to evaluate if a 2-week interruption in low-dose weekly methotrexate treatment after SARS-CoV-2 vaccine boosters enhances the immune response compared with continuing treatment in adults with autoimmune inflammatory conditions.

**Methods and analysis:**

An open-label, pragmatic, prospective, parallel group, randomised controlled superiority trial with internal feasibility assessment and nested mechanistic substudy will be conducted in rheumatology and dermatology clinics in approximately 25 UK hospitals. The sample size is 560, randomised 1:1 to intervention and usual care arms. The main outcome measure is anti-spike receptor-binding domain (RBD) antibody level, collected at prebooster (baseline), 4 weeks (primary outcome) and 12 weeks (secondary outcome) post booster vaccination. Other secondary outcome measures are patient global assessments of disease activity, disease flares and their treatment, EuroQol 5- dimention 5-level (EQ-5D-5L), self-reported adherence with advice to interrupt or continue methotrexate, neutralising antibody titre against SARS-CoV-2 (mechanistic substudy) and oral methotrexate biochemical adherence (mechanistic substudy). Analysis of B-cell memory and T-cell responses at baseline and weeks 4 and 12 will be investigated subject to obtaining additional funding. The principal analysis will be performed on the groups as randomised (ie, intention to treat). The difference between the study arms in anti-spike RBD antibody level will be estimated using mixed effects model, allowing for repeated measures clustered within participants. The models will be adjusted for randomisation factors and prior SARS-CoV-2 infection status.

**Ethics and dissemination:**

This study was approved by the Leeds West Research Ethics Committee and Health Research Authority (REC reference: 21/HRA/3483, IRAS 303827). Participants will be required to give written informed consent before taking part in the trial. Dissemination will be via peer review publications, newsletters and conferences. Results will be communicated to policymakers.

**Trial registration number:**

ISRCTN11442263.

**Strengths and limitations of this study**

- This study will recruit people with a broad range of inflammatory conditions treated with low-dose weekly methotrexate.
- It will recruit up to 560 participants and is adequately powered to detect modest differences in anti-spike receptor-binding domain antibody titres.
- It will assess both quality and quantity of the serological immune response and collect information about disease flares.
- This is an open-label study; however, primary and key secondary outcome measures are assessed by blinded laboratory staff.
- Limitations include uncertainty about the strength of the relationship between serological response and clinical outcomes, and the use of generic instruments to collect data on disease activity and flares.

## Introduction

Inflammatory conditions including rheumatoid arthritis (RA), psoriasis±arthritis, systemic lupus erythematosus (SLE) and axial spondyloarthritis affect approximately 3.5% of UK adults,^1–4^ and are associated with increased risk of COVID-19 hospitalisation and death.^5–7^ They are often treated with immune-suppressing drugs such as methotrexate, leflunomide and azathioprine.^8–10^ Of these, low-dose weekly methotrexate (≤25 mg/week) has emerged as the first-line treatment due to its efficacy, tolerability and comparative safety.^9 10^ However, methotrexate reduces antibody responses to pneumococcal polysaccharide and inactivated influenza vaccines (IIVs),^11^ and there is concern that similar effects may exist for vaccines against COVID-19. Although withholding methotrexate for 2 weeks after vaccination with the IIV increased the proportion of participants achieving protective haemagglutination inhibition (HAI) antibody titre,^12 13^ there is considerable inconsistency in advice on whether to hold or continue taking methotrexate around the time of vaccination.^14 15^

As both B-cell and T-cell responses are reduced by methotrexate, vaccinated individuals will potentially be less likely to mount a strong immune response to fight the SARS-CoV-2 infection.^16–20^ Whether a break in methotrexate treatment will improve the immune response elicited by vaccines against COVID-19 is not known. We hypothesise that individuals treated with methotrexate at the time of vaccination against COVID-19 will have an impaired immune response to the vaccine dose, and therefore lower production of anti-spike receptor-binding domain (RBD) and neutralising antibodies, and that a 2-week temporary suspension in methotrexate treatment will improve these responses without significant worsening of underlying inflammatory disease when compared with continuing with treatment as usual. Thus, the main aim of this study is to assess whether a temporary 2-week suspension of low-dose weekly methotrexate treatment immediately after SARS-CoV-2 vaccine boosters improves the vaccine response in people with inflammatory conditions, with key secondary outcomes looking at disease control. An additional mechanistic aim was to explore the efficiency of the serological response in terms of neutralisation. A sensitivity analysis of participants’ adherence to methotrexate based on a validated bioassay will be undertaken.^21^

### Objectives

#### Primary

The primary objective of this study was to assess the effectiveness of a 2-week temporary suspension of methotrexate treatment on anti-spike RBD antibody levels at 4 weeks after SARS-CoV-2 booster vaccination.

#### Secondary

The secondary objective was

- To assess the effectiveness of a 2-week suspension of methotrexate treatment on:

-  Anti-spike RBD antibody levels at week 12 post booster vaccination.
- Disease activity at weeks 2, 4 and 12 post booster vaccination.
- Disease flare-ups and their treatment during the 12 weeks post booster vaccination.
- Quality of life (QoL) at weeks 4 and 12 post booster vaccination.
- Neutralising antibody responses at weeks 4 and 12 post booster vaccination (mechanistic substudy).

- To explore association between anti-spike RBD antibody and neutralisation titres prebooster vaccination and at weeks 4 and 12 post booster vaccination (mechanistic substudy).
- To explore the validity of anti-spike RBD antibody and neutralisation titres to SARS-CoV-2 booster vaccine in participants adherent to methotrexate at each time point based on a validated biochemical assay^21^ (mechanistic substudy).

## Methods and analysis

### Study design

A two-arm, parallel group, open-label, multicentre, superiority randomised controlled trial, with 1:1 randomisation, performed in two continuous phases: a ‘pilot’ phase with evaluation of stop–go criteria at 4 months after the randomisation of the first participant, with prespecified progression criteria, followed by a main trial phase (table 1).

**Table 1:** Stop–go criteria for the Vaccine Response On/Off Methotrexate study

	Black (%)	Red (%)	Amber (%)	Green (%)
% of expected recruitment	≤25	26–50	51–75	>75
Self-reported adherence to intervention	≤40	41–60	61–80	>80
Action	Stop	Continue—major action needed in discussion with funder; protocol review, assess and resolve barriers, assess feasibility of improvement	Continue—action needed; assess and resolve barriers to recruitment/adherence	Continue—no action needed

**Figure 1:**
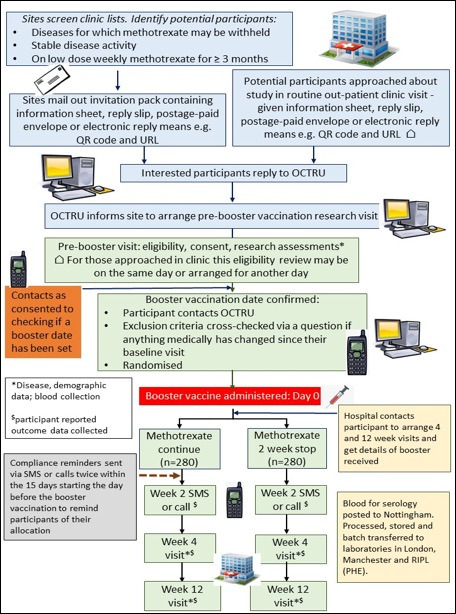
Participant flow in the Vaccine Response On/Off Methotrexate study. OCTRU, Oxford Clinical Trials Research Unit; PHE, formerly Public Health England, now UK Health Security Agency.

This trial will be conducted in approximately 25 secondary care hospitals delivering NHS provided care in England and Wales (figure 1). The research sites are a mix of district general and university hospitals. Their names can be obtained from the study website (https://vroom.octru.ox.ac.uk/vroom-home-page).

#### Recruitment

Participants will be recruited from secondary care rheumatology and dermatology clinics. The initial approach will be via a recruitment pack containing an invitation letter and a patient information sheet that participating sites will distribute to patients prescribed low-dose weekly methotrexate. Posters will also be displayed in the clinic, and participants may also be approached about the study by their usual clinical care team during consultations. Individuals wishing to take part have the option to express their interest in the study either by scanning a QR code, entering the study URL into a web browser, telephoning the study team or returning completed reply slips to the study team by post. The study opened for recruitment on 30 September 2021 and anticipates to complete recruitment by 30 June 2022.

### Eligibility criteria

#### Inclusion criteria

- Age≥18 years.
- Diagnosed with inflammatory conditions such as RA, psoriasis±arthritis, seronegative spondyloarthritis, reactive arthritis, atopic eczema, polymyalgia rheumatica and SLE. This is not an exhaustive list and people with other inflammatory conditions may also be eligible to participate in the study, provided they are able to interrupt treatment for 2 weeks as per their specialist.
- Prescribed oral or subcutaneous methotrexate (≤25 mg/week)±hydroxychloroquine for at least the previous 3 months.
- Able to temporarily suspend methotrexate for 2 weeks in the opinion of patients’ hospital team without the risk of substantial increase in disease activity, or organ or life-threatening flare-up.
- Able to give informed consent.
- Had any two vaccinations from the NHS COVID-19 Vaccination Programme between in 2020 and 2021.

#### Exclusion criteria

- Diagnosed with antineutrophil cytoplasmic antibodies **(**ANCA)-associated vasculitis, large vessel vasculitis, myositis, giant cell arteritis and solid organ transplant. This is not an exhaustive list of conditions, and if a participant is diagnosed with another inflammatory condition for which treatment cannot be interrupted safely, they will not be eligible to take part in the Vaccine Response On/Off Methotrexate (VROOM) study.
- Treated with rituximab infusion in the last 18 months or planning to start it.
- Concurrent immune suppressive treatments in the last 2 months, specifically leflunomide, ciclosporin, azathioprine or mercaptopurine, sulfasalazine or other 5-amino-salicylic acid drugs, mycophenolate, apremilast or biologics.
- Radiotherapy or cancer chemotherapy in the last 6 months.
- Prednisolone dose of >7.5 mg/day within 30 days of randomisation.
- Active solid organ cancer (people with skin cancer or those cured of solid organ cancer are eligible).
- Already participating in a clinical trial of an investigational medicinal product (CTIMP) or planning to participate in a CTIMP during the 12-week study period.

### Randomisation

Randomisation will be performed using a centralised validated computer randomisation program through a secure (encrypted) web-based service provided by the Oxford Clinical Trials Research Unit (OCTRU). Eligible participants will be randomised when they receive a date for their booster vaccination. Such participants may access the online database to complete a form which will randomise them to a treatment allocation, telephone the study team who will be able to randomise them over the telephone, or the site research team can complete the randomisation with the participant while in the clinic. At this time, the participants will be asked to confirm their consent and that their health circumstances have not changed prior to randomisation using the online interface of the study database. Those reporting a change at this stage will be referred back to the recruiting site to confirm if they are still eligible for the VROOM study. The randomisation system uses a minimisation algorithm to ensure balanced allocation across treatment groups and uses a 1:1 ratio to allocate to either continuing taking methotrexate or to have a 2-week temporary suspension immediately following their booster vaccination against COVID-19. The trial will use the following minimisation factors:

- Inflammatory condition type (inflammatory rheumatic disease (±skin disease) or skin disease alone).
- Age group (<40, 40–64 and ≥65 years).
- Previous vaccination platform received (mRNA or vector or combination).

Randomisation is being minimised on the aforementioned factors as the magnitude of immune response differs between mRNA and adenoviral vector vaccine platforms used in primary vaccination in the UK, and younger age increases the immune response to vaccines.^22^ We chose not to minimise on past COVID-19 infection even though it is a strong modifier of serological response to SARS-CoV-2 vaccines,^23–26^ as it is difficult to ascertain this reliably from participant self-report. However, we will obtain past infection status using antinucleocapsid antibodies and use this in the statistical analysis. Due to the nature of the intervention, the participants and the clinical team will not be blind to the allocated arm of the study. However, those analysing the study samples will be blinded to the participants’ allocation. Trial statisticians will not be blinded.

### Treatment arms

This trial is about temporarily suspending or continuing methotrexate treatment post vaccine booster against COVID-19 delivered by the UK’s national vaccination programme. The VROOM study will not impact on when and which booster vaccination an individual receives. If another vaccine such as IIV is also given at the same time, these data will be recorded.

#### Experimental arm

Methotrexate will be suspended for 2 weeks immediately *after* receiving the booster vaccination against COVID-19.

#### Control arm

The same dose of methotrexate will be continued as usual after having the booster vaccination against COVID-19.

In the experimental arm, if the participants are due to take their methotrexate on the day they are to receive the booster, they will be asked to refrain from taking their methotrexate on that day and then also the dose due a week later; therefore, for these individuals, it will strictly be temporarily suspending one dose on the same date as the vaccine booster and one post vaccination. In all other cases, the two doses due immediately after receiving the booster vaccination are the ones to be missed.

The study requires participants to temporarily suspend their methotrexate for 2 weeks if they are assigned to the treatment arm; any other treatments (eg, folic acid, hydroxychloroquine, prednisolone, topical treatments, etc) should be continued. Participants and their usual care team will be able to manage disease flares including with corticosteroids or any other drug as clinically appropriate. Should a clinical need arise, the participants’ usual care team will be able to advise them to interrupt or continue with methotrexate against trial allocation, for example, for infection or disease flare-up. We will collect these data but not influence the care and treatment of the participant. No concomitant care and intervention are prohibited in the trial, and the participants’ clinical care team will continue to manage their condition in the usual way after the end of their participation in the trial.

Automatic reminders by short message service (SMS) or email will be sent to participants to encourage adherence to their randomised intervention, where they consent to receive these. Participants may be telephoned by the study team if they decline the use of SMS but provide consent for reminders.

### Outcomes

#### Primary outcome

- Anti-spike RBD antibody level at 4 weeks post booster vaccination.

#### Secondary outcomes

- Anti-spike RBD antibody at 12 weeks post booster vaccination.
- Patient assessments of disease activity: global assessment using a Numerical Rating Scale (NRS) with 1-week recall at baseline, 2, 4 and 12 weeks post booster vaccination, current disease activity level and change since booster, 4 and 12 weeks post booster vaccination.
- Disease flare-up and actions taken to deal with them at 4 and 12 weeks post booster vaccination.
- Effect on QoL (assessed using EQ-5D-5L) at 4 and 12 weeks post booster vaccination.
- Adherence with advice to interrupt or continue methotrexate: self-report at 2 and 4 weeks post booster vaccination.
- COVID-19 neutralising titre (mechanistic substudy only) at 4 and 12 weeks post booster vaccination.
- Adherence to methotrexate allocation at 4 and 12 weeks post booster vaccination (mechanistic substudy only).
- T-cell and memory B-cell immune response (mechanistic substudy only) if additional funding can be secured.

#### Safety outcomes

- These include serious adverse events (SAEs) (recorded from booster vaccination to 12 weeks post vaccination).

##### Data to be collected

Data collection will occur after informed written consent is obtained by a site principal investigator or delegated member of their research team (table 2). This will include optional consent for using any leftover biological samples for additional research purposes.

**Table 2:** Vaccine Response On/Off Methotrexate study research assessments at different time points

Assessments	Prebooster (baseline)*	Booster vaccine date known†	Week after the booster date†	2 weeks post booster†	4 weeks post booster*	12 weeks post booster*
Clinical study						
Demographic	+					
Height/weight	+					
Current medications	+				+	+
Comorbidities	+					
Previous SARS-CoV-2 vaccines	+					+
Blood sample for anti-spike RBD antibody	+				+	+
Disease activity	+			+	+	+
Quality of life	+				+	+
COVID-19 disease and vaccination history	+				+	+
Disease flare-up					+	+
Randomisation		+				
Reminder of allocation and to continue or withhold methotrexate		+	+			
Adherence to intervention				+	+	
Safety				+	+	+
Details of vaccination					+	
Mechanistic						
Blood sample taken for neutralisation assay‡	+				+	+
Blood sample taken for methotrexate adherence bioassay‡	+				+	+
Blood sample taken for T-cell and B-cell responses§	+				+	+

*Face to face in clinic, must take place a minimum of 6 weeks from prior vaccination against COVID-19.

†Remote via text, email or phone call.

‡In a subset of 100 participants.

§In a subset of participants where recruiting site is able to take and extract peripheral blood mononuclear cells (PBMCs). These will be analysed once additional finding is obtained.

RBD, receptor-binding domain.

###### Baseline visit

Data on demographic factors (age, sex, ethnicity, usual residence (home or residential care)); smoking status; inflammatory conditions; self-reported physician diagnosis of diabetes including diet-controlled diabetes, hypertension, ischaemic heart disease, congestive cardiac failure, asthma, chronic obstructive pulmonary disease, high cholesterol, stroke including transient ischaemic attack; current use of concomitant systemic corticosteroids, hydroxychloroquine, antidiabetic drugs and folic acid; COVID-19 disease and vaccination history; methotrexate dose, route and day of administration; and dose of hydroxychloroquine (if taken) will be self-reported. QoL will be assessed using EQ-5D-5L. Patient global assessment of disease activity will be assessed on a 0–10 NRS for the past week using the question ‘In all the ways that your condition affects you, over the last 7 days, how would you rate the way you felt?’

Research nurses will measure height and weight and record the serum creatinine and albumin from the latest available hospital records.

###### Two weeks post booster (+5 days)

Information on adherence to the intervention and patient global assessment of disease activity will be collected, the latter using the same questions asked at the baseline visit. These data will be preferentially collected using a link to the online Research Electronic Data Capture (REDCap) survey sent in a text message, or by email if the participant prefers not to use a mobile phone for this purpose. Where an individual prefers not to receive this survey link by email or text, this information will be collected by telephone calls instead. For those who do not reply to the 2-week questions, adherence to their allocation will be checked at the 4-week visit.

**Week 4 and 12 post booster vaccination (±10 days)**

Data on the date of booster vaccination, brand of booster vaccine against COVID-19 received and administration of other vaccinations (eg, IIV) at the same time will be collected from the participant at the week 4 visit. Participants will also be requested to self-report prior pneumococcal vaccination alongside the month and year of vaccination at week 12. Confirmation of protocol compliance and information on dose, route and day of methotrexate taken in weeks 3 and 4 will be collected at the week 4 visit. Information on methotrexate dose, route and day of administration will also be collected at the week 12 visit. In addition to this, information on use of concomitant systemic medications, QoL, self-report of disease activity, information about disease flares and their treatment, COVID-19 illness and any SAEs possibly, probably or definitely related to the study intervention will be collected at week 4 and 12 visits using the same questions as at the baseline visit. Information on any further booster vaccinations against COVID-19 received since the week 4 visit will be ascertained at week 12.

###### Sample collection and transport

Seven-millilitre blood will be collected in serum separator tubes at baseline and week 4 and 12 visits. These samples will be transported to a central laboratory in the University of Nottingham in Royal Mail Safe Boxes. The central laboratory will centrifuge the samples on the day of arrival, aliquot in cryovials and store at −80°C. They will be sent on dry ice to the laboratories conducting the analyses.

#### Laboratory analyses

- Anti-spike RBD (primary endpoint at 4 weeks) and nucleocapsid antibodies: Antibody measurements will be undertaken at the UK Health Security Agency (formerly Public Health England) Rare & Imported Pathogens Laboratory using validated commercial assays ROCHE-S and ROCHE-N for anti-spike RBD and antinucleocapsid antibodies respectively.^27 28^
  ROCHE S refers to the Roche Elecsys Anti-SARS-CoV-2 S immunoassay for the in vitro quantitative determination of antibodies (including IgG) to the SARS-CoV-2 spike protein RBD. The assay uses a recombinant protein representing the RBD of the S antigen in a double-antigen sandwich assay format, which favours detection of high affinity antibodies against SARS-CoV-2.
  ROCHE N refers to the Roche Elecsys Anti-SARS-CoV-2 assay. It uses a modified double-antigen sandwich immunoassay using recombinant nucleocapsid protein (N), which is geared towards the detection of late, mature, high-affinity antibodies independent of the subclass. It is a total SARS-CoV-2 antibody assay (IgA, IgM and IgG) detecting predominantly, but not exclusively, IgG.
- Neutralising antibody titres: Neutralisation assays with authentic live virus (Wuhan Hu-1 SARS-CoV-2) and any other strains of interest will be performed as described earlier.^29^ All experiments will be conducted in duplicate and absorbance readings will be standardised against positive and negative controls, and averaged. Neutralisation curves will be plotted, with the percentage neutralisation modelled as a logistic function of the serum dilution factor (log10). A non-linear regression (curve fit) method will be used to determine the dilution fold that neutralised 50% (IC50) of the samples.
- Methotrexate biomarker: This biochemical assay uses liquid chromatography–tandem mass spectrometry performed on a Waters TQ-S Micro Triple Quadrupole Mass Spectrometry.^21^ It provides an objective measurement of adherence and has been developed with drug concentration limits according to methotrexate dose and detects methotrexate partial omission or delayed ingestion. Adherence, defined as ingestion of methotrexate in the past 6 days, is dichotomised.

#### Data protection and confidentiality

Personal information about potential and enrolled participants will be collected and processed securely, in compliance with the Data Protection Act (DPA) and General Data Protection Regulation (GDPR).

### Sample size and justification

#### Main trial

A total of 560 participants will be randomised. The sample-size estimates were derived from Folegatti *et al*, which allow the mean (SD) of the anti-spike IgG 28 days after vaccination to be estimated as 191.9 (165.5) ELISA units.^30–32^ The sample size was based on detecting at least a 25% lower antibody response in the methotrexate continuation group (Cohen’s d effect of 0.29) with 90% statistical power at a two-sided 5% significance level which requires data from 502 participants. Subsequent to starting the trial, the Roche-S assay was adopted for determining the primary outcome, given its widespread usage and performance.^27 28^ Using Roche-S anti-RBD data for healthcare workers receiving further vaccine doses,^33^ the target effect size translates to a target difference in this anti-spike RBD antibody titre of around 5000. After allowing for up to 10% missing data, the required sample size of 560 was chosen. This calculation was performed using Stata v.15.1.

***Rationale for choosing 25% difference in anti-spike RBD antibody level for sample-size calculation:***

Initial studies indicated that anti-spike RBD antibody might emerge as a potential correlate of protection^34^ from COVID-19. There is a correlation with viral neutralisation titres,^24 26 30 33–38^ the strength of which depends on the variant of concern being tested.^26 33^ Interrupting methotrexate for 2 weeks improved the titre of antibodies against H1N1, H3N2, B-Yamagata and B-Victoria strains in the quadrivalent influenza vaccine by 59%, 92%, 50% and 68%, respectively.^13^ Taking this into account, and given the lack of certainty around the serological correlate of protection from COVID-19 alongside a higher risk of serious complications than with seasonal influenza, the study is powered for detecting a much smaller difference. A <25% difference in anti-spike RBD antibody is unlikely to be of immediate clinical relevance, given the fact that most vaccines against COVID-19 have a very high level of protection from complications of SARS-CoV-2 infection.^30 34 35 39–41^ However, a 25% lower anti-spike RBD titre may result in 45 days’ shorter protection from infection or severe COVID-19 using the half-life estimated by Dan *et al*.^42^

#### Mechanistic substudy

This will be performed in 100 randomised participants with samples at baseline and weeks 4 and 12 post COVID-19 booster vaccination. This will enable detection of a difference between treatment arms of 0.6 SD with 80% power, 5% significance level and allowing 10% loss to follow-up; this corresponds to increases in the methotrexate interruption arm compared with the control arm of 54% (48.2) for neutralisation assay using pseudoviruses, based on an observed mean of 91.0 (SD 81.6).^30^ These differences are similar to the increase in HAI antibody titres observed with the quadrivalent influenza vaccine.^13^

### Patient and public involvement (PPI)

Two PPI meetings with eight people with lived experience of inflammatory conditions and many taking methotrexate were held in March 2021. All patient and public volunteers felt that the study was ‘definitely worth’ conducting. They felt that adherence to the intervention will be excellent and that the intervention, that is, a 2-week break in treatment, was acceptable to them as it offered the best balance of potential benefit without risking a disease flare that could happen with a longer (eg, 4-week) treatment pause. Many had paused methotrexate for 2 weeks, for example, prior to surgery or during an infection without their condition flaring up. The patient research partners supported the use of antibodies as the primary outcome but also advised to include outcomes to assess self-reported disease activity, flares and side effects. Given the broad eligibility criteria, the PPI volunteers supported the use of a few questions covering all conditions rather than using a different set of questions for each condition.

Patients will not be involved in the recruitment to and conduct of the study. However, patient partners will assist in the interpretation of overall study findings and communication to the general public. Where appropriate, patient advisors will be coauthors on publications. We will work with our PPI collaborators to ensure any plain English parts of the monograph are written in truly plain English. A post-trial dissemination event will be held inviting PPI members.

### Statistical analysis

Full details will be presented in a separate statistical analysis plan which will be drafted early in the study, finalised prior to the interim analysis data lock, and will receive review and input from the trial steering committee (TSC) and data monitoring committee (DMC). The principal analysis will be performed on the as randomised (‘intention-to-treat’) population, analysing participants with available outcome data in their randomised groups, regardless of adherence. The study will be reported in line with Consolidated Standards of Reporting Trials guidelines.

The primary objective of the statistical analysis was to identify if a temporary 2-week suspension of methotrexate after the booster vaccination against SARS-CoV-2 increases the anti-spike RBD antibody at 4 weeks post booster vaccination compared with continuing methotrexate. The differences between the study arms will be estimated a using a multilevel mixed effects regression model, allowing for repeated measures clustered within participants. The model will be adjusted for randomisation factors (inflammatory condition, age categories, prime vaccine’s platform, ie, mRNA vs other), prior infection status obtained from prevaccination antinucleocapsid antibodies and type of SARS-CoV-19 vaccine booster received as fixed effects. A treatment by time interaction will be included. The model is anticipated to use an unstructured covariance matrix and maximum likelihood estimation. Data will be log-transformed prior to analysis, as appropriate. Model diagnostics, including approximate normality of the residuals, will be assessed. A simpler model (linear regression adjusted for the randomisation factors) will be used in the event of convergence problems. Adjusted mean differences between the groups will be presented, together with 95% CI and p values.

Consistency of the treatment effects for important prognostic subgroups (methotrexate dose, inflammatory condition type, age group, previous SARS-CoV-2 infection, prime vaccination platform, booster platform, number of prior vaccination doses and route of administration of methotrexate: subcutaneous vs oral) will be explored with 95% CIs. The subgroup effects will be obtained from linear models for the 4-week primary outcome, adjusted in line with the aforementioned model specifications, and an interaction between randomised treatment and subgroup. We will also explore the time effect of delay between the original vaccination and the booster. Findings will be presented graphically and viewed as exploratory. The effect of non-compliance to the randomised intervention will be explored using per-protocol and complier-average causal effects analyses. Similar analyses will be performed for week 12 data. Secondary outcomes will be analysed using generalised linear models for binary and continuous data, as appropriate, with model adjustment as described previously.

The number of SAEs will be presented by treatment arm. The proportion of participants with at least one SAE will be compared. Details of the events, including expectedness and relatedness of the SAEs, will be presented, together with information on the timing of the events.

Further analyses related to the mechanistic hypotheses will be carried out exclusively on the mechanistic subsample including quantifying levels and strength of relationships using appropriate statistical summaries (mean, SD, range, correlation coefficients, etc). Neutralising antibody titres will be compared between the two groups at different time points using parametric or non-parametric tests, depending on data distribution. Additionally, the proportion of samples with IC50 greater than 49 will be compared between the two groups as this has been shown to prevent clinical infection in rhesus macaques.^37^ Other analyses will look at proportion with titres above 200. Sensitivity analysis for primary and key secondary outcomes will be conducted in participants adherent to methotrexate using validated biochemical measurement.^21^

Missing data will be described with reasons given where available; the number and percentage of individuals in the missing category will be presented by intervention arm.

Sensitivity analyses will be undertaken to assess the underlying missing data assumptions. The effect of deviations from the missing at random assumption made in the primary analysis will be explored by considering a range of plausible missing not at random scenarios, whereby participants with missing outcomes will be assumed to have worse outcomes than participants with available data. These sensitivity analyses will be implemented using pattern mixture models using Stata’s ‘rctmiss’ command or similar.

### Timing of analysis

The final unblinded (to the study’s non-statistician investigators) statistical analysis will take place after all follow-ups have been completed, and sufficient time has been allowed for data collection and cleaning. A single interim analysis is planned to take place once primary outcome data are available for 250 participants. Decisions for stopping the trial early for benefit or futility will be based on a Haybittle-Peto stopping boundary (p≤0.001 for the primary endpoint), but also taking account of the representativeness of the study population, magnitude of estimated effect, sufficient participants having been recruited into the important subgroups, sufficient data being available for the mechanistic components of the study and attrition. The independent DMC will be responsible for making a recommendation to the TSC. The trial statisticians will have access to the final trial dataset. Once the study has been completed and the main findings have been published, the Chief Investigator will also have access to the final trial dataset.

### Data monitoring, study management and protocol changes

Details of the study monitoring procedures (including the DMC and auditing), study management and protocol changes are provided in online supplemental file 1.

10.1136/bmjopen-2022-062599.supp1 Supplementary data

## Ethics and dissemination

This study has been approved by Leeds West Research Ethics Committee and Health Research Authority (REC reference 21/HRA/3483, IRAS 303827). Participants will be required to give written informed consent before taking part in the trial (online supplemental file 2). It will be publicised to research, clinical and patient communities and other important stakeholders, such as self-help groups.

10.1136/bmjopen-2022-062599.supp2 Supplementary data

Once the study is completed, in addition to the final report for the NIHR Efficacy and Mechanism Evaluation programme, we aim to publish the study results in peer-reviewed high-impact journals and present at national and international meetings to ensure maximum impact and rapid dissemination. Additionally, we will seek to disseminate findings through publication in other journals, such as *Pulse*, newsletters to the British Society for Rheumatology, British Association of Dermatology and Royal College of General Practitioners. We will engage with international rheumatology and dermatology societies and disseminate our results widely to change health policy at international level. The results of this study will also provide the Joint Committee on Vaccination and Immunisation and specialist societies with the requisite evidence base to recommend continuing or temporarily suspend methotrexate after SARS-CoV-2 vaccine boosters. The study’s PPI volunteers will advise on the content of all public-facing content for dissemination. There will be no restrictions on the publication of study findings. All authors will be required to meet the International Committee of Medical Journal Editors (ICJME) authorship requirements. Professional writers will not be used in the study reporting. Participant-level dataset and statistical code will be made available on reasonable request to OCTRU and the CI once the VROOM study findings have been published in full. Some specific data items may not be shared in order to maintain participant anonymity. The full study protocol may be accessed from the NIHR website (https://fundingawards.nihr.ac.uk/award/NIHR134607).

## Discussion

The VROOM study is designed to investigate whether a 2-week break in low-dose weekly methotrexate will improve the immune response elicited by vaccines against COVID-19 in people with inflammatory conditions taking methotrexate for at least the previous 3 months. It will evaluate both the quantity and quality of the antibody response. It has broad eligibility criteria and the study results will be generalisable to common inflammatory conditions for which treatment may be interrupted safely. Nevertheless, such treatment interruptions carry the risk of disease flare-ups, and data on this outcome will be collected to provide a complete picture of the risks and benefits of this strategy. The relatively large sample size will allow us to conduct several a priori subgroup analyses. We anticipate that the results of this study will inform treatment decisions around the time of future boosters against vaccination against COVID-19.

Given the broad eligibility criteria, it is not possible to use disease-specific outcome measures for measuring disease activity. Thus, we have chosen to use global disease activity using NRS and QoL using EQ-5D-5L in this study. This is a potential limitation. Other key limitations are uncertainty about the strength of the relationship between antibody response and clinical outcomes such as severe infection and mortality. Due to non-blinding of the participants, there is greater potential for non-compliance with the allocation. Our primary endpoint is though objective and will be analysed by blinded assessors in a central laboratory, and we will measure compliance with the advice to hold or continue treatment in both arms and to assess its effect in a prespecified sensitivity analysis.

Results of the VROOM study will reduce uncertainties on whether people on long-term low-dose methotrexate should hold their treatment for a 2-week period after vaccination against COVID-19 to improve their immune response. The results will inform national and international treatment recommendations. It may serve as a template for future similar trials for other medicines.

## Supplementary Material

Reviewer comments

## Data Availability

Data are available upon reasonable request. Participant-level dataset and statistical code will be made available upon reasonable request to the Oxford Clinical Trials Research Unit and the CI, once the Vaccine Response On/Off Methotrexate study findings have been published in full. Some specific data items may not be shared in order to maintain participant anonymity.
