# Supplementary material for: Effects of temporarily suspending low-dose methotrexate treatment for 2 weeks after SARS-CoV-2 vaccine booster on vaccine response in immunosuppressed adults with inflammatory conditions: protocol for a multicentre randomised controlled trial and nested mechanistic substudy (Vaccine Response On/Off Methotrexate (VROOM) study)
Source: BMJ Open. 2022 May 3;12(5):e062599. doi: 10.1136/bmjopen-2022-062599 (PMC9066090; doi:10.1136/bmjopen-2022-062599)
Supplement: Supplementary data [file bmjopen-2022-062599supp001.pdf]

Supplementary material

1. Monitoring

2. Summary of protocol changes

1. Monitoring

Data Monitoring Committee (DMC) A DMC comprising of a group of three independent experts including at least one statistician and one clinician, external to the study will be constituted. It will assess the progress, conduct and critical outcomes of the study and will meet regularly throughout the study at time-points agreed by the Chair of the Committee and the CI. The DMC will review the safety data generated, including all serious adverse events, and make recommendations as to whether the protocol should be amended to protect patient safety. It will report to the chair of the TSC via the trial statistician. The DMC is independent of the sponsor and will maintain a list of conflicts of interest.

Auditing: The Oxford Clinical Trials and Research Unit (OCTRU) Quality Assurance team will undertake periodic audits on behalf of the sponsor.

Study management

The Chief Investigator has overall responsibility for the study and shall oversee all study management. The data custodian will be the Chief Investigator.

Trial Steering Committee (TSC) The role of the independent TSC is to provide the overall supervision of the study. They will monitor the study's progress and conduct, and will advise on scientific credibility. The committee will consider and act, as appropriate, upon the recommendations of the DMC and ultimately carries the responsibility for deciding whether the study needs to be stopped on grounds of safety or efficacy. The committee includes independent members and members of the research team.

Trial Management Group (TMG) consists of those individuals responsible for the operational management of the study such as the chief and -co-investigator, EMR operational lead, the trial manager and the trial statistician. Other specialities/ individuals will be invited as required for specific items/issues.

The TMG will meet usually at least once a month throughout the lifetime of the study and will:

- Supervise the conduct and progress of the study, and adherence to the study protocol;
- Assess the safety as compiled by the study team and assessed by the DMC;
- Evaluate the quality of the study data.

2. Summary of protocol changes

Protocol changes will be communicated to the funders, study steering committees, and trial sites as necessary by email.

V1.0	First edition of protocol reviewed by the REC – note no participants were recruited under this version
V2.0	Typographic changes including adding in Free phone number, addition of ISRCTN details, addition of exclusion of planning to participate in a CTIMP during the 12-week study period, removal of 4-month maximum time period between baseline data collection and booster vaccination. Addition of permitted time window around 2-week visit.
V3.0	Addition of ability for some sites to take additional blood samples to allow for extraction and storage of Peripheral Blood Mononuclear Cells (PBMCs) for potential laboratory analysis on T and B Cells if operational and scientific capacities allow. Addition of taking COVID history at weeks 4 & 12.

V4.0	Eligibility criteria broadened to allow recruitment of patients around the time of their 3rd or 4th booster. Introduction of a minimum time period between a COVID-19 vaccination and a baseline appointment. Addition of a question at 12-week time point regarding whether a pneumococcal vaccine or any further COVID-19 vaccines have been received. Clarification of sample size calculation.
V5.0	Change of anti-spike-RBD immunoassay from Euroimmun to Roche Elecsys® Anti-SARS-CoV-2 S immunoassay. Clarification of exclusion criteria.

BMJ Publishing Group Limited (BMJ) disclaims all liability and responsibility arising from any reliance placed on this supplemental material which has been supplied by the author(s)
