# Supplementary material for: Effects of temporarily suspending low-dose methotrexate treatment for 2 weeks after SARS-CoV-2 vaccine booster on vaccine response in immunosuppressed adults with inflammatory conditions: protocol for a multicentre randomised controlled trial and nested mechanistic substudy (Vaccine Response On/Off Methotrexate (VROOM) study)
Source: BMJ Open. 2022 May 3;12(5):e062599. doi: 10.1136/bmjopen-2022-062599 (PMC9066090; doi:10.1136/bmjopen-2022-062599)
Supplement: Supplementary data [file bmjopen-2022-062599supp002.pdf]

PARTICIPANT CONSENT FORM – IRAS ID: 303827

A multi-centre open-label two-arm randomised clinical trial of Vaccine Response On/Off Methotrexate (VROOM)

If you agree, please initial box
(In the electronic version of the form – individuals will select a Yes option instead of initials against each clause)

1. I confirm that I have read the information sheet dated 09September2021 (version 3.0) for this study. I have had the opportunity to consider the information, ask questions and have had these answered satisfactorily.		
2. I understand that my participation is voluntary and that I am free to withdraw at any time without giving any reason, without my medical care or legal rights being affected. I understand that should I withdraw then the information collected so far cannot be erased and that this information may still be used in the project analysis.		
3. I understand that relevant sections of my medical notes and data collected during the study may be looked at by authorised individuals from Universities of Nottingham and Oxford, from regulatory authorities and from the NHS Trust(s), where it is relevant to my taking part in this research. I give permission for these individuals to have access to these records and to collect, store, analyse and publish information obtained from my participation in this study. I understand that my personal details will be kept confidential.		
4. I agree to my General Practitioner (GP) being informed of my participation in this study.		
5. I understand that the information collected about me will be used to support other research in the future, and may be shared anonymously with other researchers		
6. I agree to my personal contact details being recorded and used for the purpose of this study.		
7. I understand and agree that blood samples will be taken for analysis of anti-spike RBD antibody (and for a subset of participants – for levels of methotrexate and neutralising antibodies)		
**8. Consent for storage and use in possible future research (Optional)** I agree that the samples I have given and the information gathered about me can be stored by the University of Nottingham at the Clinical Sciences Building on the City Hospital Campus, for possible use in future studies. I understand that some of these studies may be carried out by researchers other than the current team who ran the first study, including researchers working for commercial companies. Any samples or data used will be anonymised, and I will not be identified in any way.	Yes	No
9. I agree to take part in the VROOM study.		

Abhishek A, *et al. BMJ Open* 2022; 12:e062599. doi: 10.1136/bmjopen-2022-062599

BOOSTER DATE

Please select which option you would like to use to be reminded about letting the VROOM study team know when you get a date for your booster

10. Whilst waiting for my booster vaccination: (select one option)	
I agree to receive **weekly text (SMS) messages.**	
I agree to receive **weekly short telephone calls.**	
I agree to receive **weekly emails.**	
I do not want to be contacted to check if I have received my COVID-19 booster appointment – I will contact the VROOM study team when I get my booster appointment date.	

AFTER YOUR BOOSTER VACCINATION

Please select which option you would like to use to immediately after your booster:

11. Immediately after my booster vaccination in the 2 weeks after it – in order to remind you of the group you are in and to find out how you are: (please select one option)	
I agree to receive up to **6 text (SMS) messages** from the VROOM study team.	
I agree to receive up to **6 short telephone calls** from the VROOM study team.	
I agree to receive up to **6 emails** from the VROOM study team.	

4 and 12 WEEKS AFTER YOUR BOOSTER VACCINATION

Please select which option you would like to use in the 12 weeks after your booster regarding the study questionnaire pack (which should take no more than 10 minutes):

12. Questionnaire pack completion – please select one option:	
I agree for the questionnaires to be sent to me ahead of returning to the hospital by **email**.	
I agree for the questionnaires to be sent to me ahead of returning to the hospital by **post.**	
I will wait to complete the questionnaires when I return for my hospital visit.	

AT THE END OF THE STUDY

13. At the end of the study – how would you like a copy of the study and your blood sample results: (please select one option)	
I agree to receive the results by **email.**	
I agree to receive the results by **post.**	

Name of participant ________ Date ________ Signature ________

Name of person taking consent ________ Date ________ Signature ________

Participant registration ID: ______________________

Abhishek A, *et al. BMJ Open* 2022; 12:e062599. doi: 10.1136/bmjopen-2022-062599
