# Supplementary material for: Effects of temporarily suspending low-dose methotrexate treatment for 2 weeks after SARS-CoV-2 vaccine booster on vaccine response in immunosuppressed adults with inflammatory conditions: protocol for a multicentre randomised controlled trial and nested mechanistic substudy (Vaccine Response On/Off Methotrexate (VROOM) study)
Source: BMJ Open. 2022 May 3;12(5):e062599. doi: 10.1136/bmjopen-2022-062599 (PMC9066090; doi:10.1136/bmjopen-2022-062599)
Supplement: Reviewer comments [file bmjopen-2022-062599.reviewer_comments.pdf]

## PEER REVIEW HISTORY

BMJ Open publishes all reviews undertaken for accepted manuscripts. Reviewers are asked to complete a checklist review form (**http://bmjopen.bmj.com/site/about/resources/checklist.pdf**) and are provided with free text boxes to elaborate on their assessment. These free text comments are reproduced below.

## ARTICLE DETAILS

| | |
|---|---|
| **TITLE (PROVISIONAL)** | Effects of temporarily suspending low-dose methotrexate treatment for two weeks after SARS-CoV-2 vaccine booster on vaccine response in immunosuppressed adults with inflammatory conditions: protocol for a multicentre randomised controlled trial and nested mechanistic sub-study (Vaccine Response On/Off Methotrexate [VROOM] study) |
| **AUTHORS** | Abhishek, Abhishek; Boyton, RJ; McKnight, Áine; Coates, Laura; Bluett, James; Barber, Vicki; Cureton, Lucy; Francis, Anne; Appelbe, Duncan; Eldridge, Lucy; Julier, Patrick; Peckham, Nicholas; Valdes, A; Rombach, Ines; Altmann, Daniel; Nguyen-Van-Tam, Jonathan; Williams, HC; Cook, Jonathan |

This article was not externally reviewed at BMJ Open. Protocols that have been independently assessed prior to submission to BMJ Open are usually fast-tracked to publication on the grounds that further substantial changes will not be possible. This independent assessment will usually be external, independent review for both a substantial grant award from a non-commercial or government funder and ethics approval.
